# Morning Sleep Inertia in Alertness and Performance: Effect of Cognitive Domain and White Light Conditions 

**DOI:** 10.1371/journal.pone.0079688

**Published:** 2013-11-18

**Authors:** Nayantara Santhi, John A. Groeger, Simon N. Archer, Marina Gimenez, Luc J. M. Schlangen, Derk-Jan Dijk

**Affiliations:** 1 Surrey Sleep Research Centre, Faculty of Health and Medical Sciences, University of Surrey, Guildford, United Kingdom; 2 Department of Psychology, University of Hull, Hull, United Kingdom; 3 Philips Research, Eindhoven, The Netherlands; Federal University of Rio de Janeiro, Brazil

## Abstract

The transition from sleep to wakefulness entails a temporary period of reduced alertness and impaired performance known as sleep inertia. The extent to which its severity varies with task and cognitive processes remains unclear. We examined sleep inertia in alertness, attention, working memory and cognitive throughput with the Karolinska Sleepiness Scale (KSS), the Psychomotor Vigilance Task (PVT), n-back and add tasks, respectively. The tasks were administered 2 hours before bedtime and at regular intervals for four hours, starting immediately after awakening in the morning, in eleven participants, in a four-way cross-over laboratory design. We also investigated whether exposure to Blue-Enhanced or Bright Blue-Enhanced white light would reduce sleep inertia. Alertness and all cognitive processes were impaired immediately upon awakening (p<0.01). However, alertness and sustained attention were more affected than cognitive throughput and working memory. Moreover, speed was more affected than accuracy of responses. The light conditions had no differential effect on performance except in the 3-back task (p<0.01), where response times (RT) at the end of four hours in the two Blue-Enhanced white light conditions were faster (200 ms) than at wake time. We conclude that the effect of sleep inertia varies with cognitive domain and that it’s spectral/intensity response to light is different from that of sleepiness. That is, just increasing blue-wavelength in light may not be sufficient to reduce sleep inertia. These findings have implications for critical professions like medicine, law-enforcement etc., in which, personnel routinely wake up from night-time sleep to respond to emergency situations.

## Introduction

 We wake up consciously aware but rarely fully alert. Awakening from sleep entails a temporary period of reduced alertness and impaired cognition known as sleep inertia. First described in the early 1960’s [1], it’s effects on performance, like sleep loss, includes a slowing of responses, loss of accuracy, increased attentional lapses and reduced alertness [2]. Whether this means that sleep inertia is a part of the sleepiness continuum driven by the homeostatic process remains an unresolved issue. In most current models of alertness and performance sleep inertia is conceptualized as a separate physiological state distinct from sleepiness [3–5]. 

Why sleep inertia occurs is not clear. It has been postulated that a temporal dissociation between two wake-up processes, one, a rapid restoration of conscious awareness and the other, a slower more progressive recovery to full alertness, underlies the phenomenon [6]. This speculation by Balkin et al. [6] is based on their brain imaging study, in which, immediately after awakening, areas such as the brainstem, basal ganglia and thalamus appeared to be rapidly maximally reactivated and remained stable thereafter, whereas, anterior cortical areas associated with higher order cognitive functions continued to increase in activation for up to fifteen minutes post-awakening. More recently, Groeger et al. [15] provided additional support for this notion. They found that in sleep restricted participants awakening from an afternoon nap, executive functions took longer to recover to baseline levels than performance in simple tasks. 

Sleep inertia is strongest at wake-time, dissipating rapidly thereafter. Estimates of the dissipation range from a minute to over two hours [3,4,7,8] and this has been attributed to a number of factors including prior sleep duration [9], sleep stage at awakening [10–12], circadian phase at awakening [13], and even chronotype [14]. Crucially, a differential sensitivity of various cognitive processes to sleep inertia may be a significant contributing factor, but this has not been thoroughly investigated. To do so, it is necessary to compare performance in several tasks that differ appropriately, e.g. 1- back vs. 3-back (for executive function load in working memory) [15]. In fact, using such an approach, Groeger et al. [15] showed that executive loading worsens the impact of sleep inertia following afternoon naps but not morning naps. However, with a few exceptions [15–17], most studies have focused on subjective measures of alertness and a single cognitive task [1,3,4,7,8,18]. 

 Although, sleep inertia is a robust phenomenon with obvious practical consequences, strategies to counteract it remain undeveloped. Given the fact that light has a powerful non-visual effect on behaviour, and, that brain activation while people are engaged in a cognitive task can be enhanced by light exposure [19,20], one wonders whether artificial light can be engineered to counteract sleep inertia. In this regard, blue-enhanced light may be an attractive candidate given that it enhances alertness and improves response times in simple cognitive tasks [21–23]. Also, given that changes in temperature and cortisol are associated with differential cognitive performance [24,25], the fact that simulated artificial dawn light affects temperature [8] and cortisol [26] is encouraging. 

 We examined morning sleep inertia, concurrently, in several cognitive processes and investigated whether increasing the level of blue wavelength in artificial white light can counteract sleep inertia. We measured subjective sleepiness, sustained attention, cognitive throughput and working memory with the Karolinska Sleepiness Scale (KSS), the Psychomotor Vigilance Task (PVT), the add task and the verbal 1 and 3-back tasks, respectively [15,27–29], under four white light conditions, each with a different spectral and intensity characteristic. Sleep inertia affected sleepiness and all the cognitive processes tested, with the impact being strongest in subjective sleepiness. However, increasing blue wavelength in white light had little effect on sleep inertia with the exception of the 3-back task. 

## Materials and Methods

### Ethics Statement

 This study was conducted in accordance with the principles expressed in the Declaration of Helsinki. The protocol was approved by the University of Surrey Ethics Committee (EC/2011/102/FHMS) and written informed consent was obtained from all participants prior to the start of any study related procedures. 

### Participants

 Sixty-two healthy individuals aged 18 to 35 were screened into the study, of whom twelve were invited as participants and two as reserves. All fourteen were healthy by history, physical examination (including the Ishihara test for color blindness) and standard biochemistry/haematology. They had regular self-reported sleep/wake patterns and were not on any medication. Self-reported need for at least 60 minutes to feel fully alert after awakening was a primary selection criteria [18]. Exclusionary criteria included recent history of shift-work, travel across more than one time-zone in the preceding twelve months, Pittsburgh Sleep Quality Index (PSQI) score > 5 [30], consumption of more than 4 cups of caffeinated beverages per day and/or 14 units of alcohol per week and a positive urinary toxicology screen for substances of abuse. Three participants withdrew early in the protocol and were replaced by the two reserves, resulting in eleven (7 females; mean age +/- SD: 22.27 +/- 4.22 years) completing the protocol. Additionally, at screening, buccal swab samples were collected for extracting genomic DNA to analyse for the Variable Number Tandem Repeat (VNTR) polymorphism in the clock gene *PERIOD3* (*PER3*), which is associated with diurnal preference [31,32]. But, analyses based on this polymorphism are not presented here.

### Protocol

 The experiment was conducted in the UK (51°14'07.44"N latitude), between October and December, inclusive. 

#### Pre-Laboratory Segment

For a week to ten days before the first laboratory session, participants wore an actiwatch (Actiwatch Spectrum, Philips Respironics) and filled out sleep diaries (Karolinska Sleep Diary and Social Rhythm Metric scale). This allowed us to determine their habitual sleep-wake schedule and daily light exposure pattern [33,34]. Following this, a week prior to each laboratory visit, participants were assigned an 8-hour sleep schedule (which was within an hour of their habitual bedtime). Their compliance was verified with actigraphy monitoring and sleep diary. Actigraphy data (namely, the hourly time bin in which an onset and offset threshold activity occurred) indicated they all maintained this schedule to within 40 minutes of their assigned bedtime and wake-time.

#### Laboratory Segment

**Figure 1:**
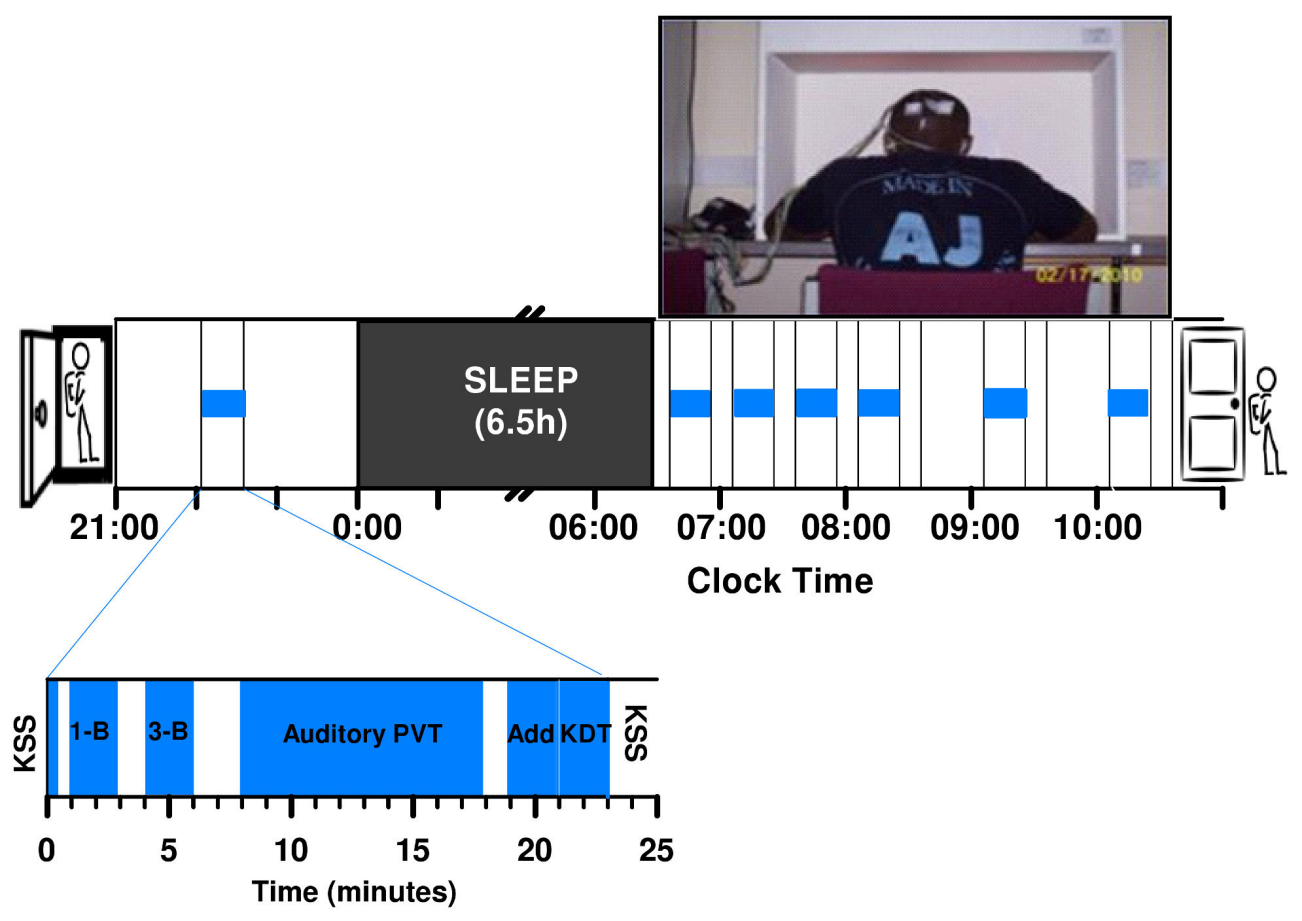
Protocol. The timing of events in each laboratory session occurred as shown in the figure. Baseline assessment preceded the 6.5 hour night time sleep episode, which was followed by the sleep inertia assessment conducted during a four hour light exposure as shown in the top right box of the figure. The the timing and order of administration of the cognitive tasks in both baseline and sleep inertia assessments occurred as shown in the bottom left rectangle. The light box measuring 138 x 80 x 90 cm and painted white on the inside, contained two top-mounted commercially available fluorescent tubes (Philips T5 24W lamps 827: 2700K light; Activiva Active: 17000K light with a Color Rendering Index > 80). A diffuser covered the tubes to yield a diffuse relatively homogeneous light distribution within the box. Consent to publish the photograph of the light box was obtained.

The five laboratory sessions spanned 6 weeks with a week between each session. The first, an *acclimation* session, included polysomnographic (PSG) recording of night-time sleep and two hours of cognitive testing after awakening in the morning. This cognitive session served to introduce the different tasks to the participants and provide them an opportunity to reach asymptotic levels of performance to minimize practice effects [35]. To preserve equivalence across laboratory sessions, the acclimation cognitive testing set-up included the light box (Figure 1), but without light exposure. The four *experimental* sessions included overnight sleep (*6.5 hrs long* to optimise sleep inertia [9]) in the laboratory, preceded by baseline cognitive assessment and followed by sleep inertia assessment during a four-hour morning light exposure (Figure 1). All laboratory events were timed relative to a study designated bedtime (00:12, 00:37 and 00:57). Having these three staggered bedtimes enabled us to maintain precise timing of cognitive testing in multiple participants, while at the same time minimizing deviations from the subjects’ habitual bedtime (average +/- 1SD: 23:23 +/- 00:42). 

 On the day of the experimental session, participants arrived at the Surrey Clinical Research Centre (SCRC) between 19:00 - 19:30 h. Upon arrival, they underwent a basic medical screen and alcohol breathalyzer test, followed by electrode application and baseline cognitive assessment (conducted two hours before lights-out in 10-20 lux ambient white light). In the morning, within three minutes of lights-on (Figure 1) we started the light exposure with multiple sleep inertia assessments (identical to baseline assessment). Measurements included actigraphy, waking electroencephalography (EEG) with half-hourly administrations of the Karolinska Drowsiness Test (KDT; not discussed here), KSS (half-hourly), cognitive testing (half hourly during the first 90 minutes of light exposure and hourly thereafter) and a headache questionnaire (not discussed here). After the last cognitive assessment, participants received a small snack. At the end of the light exposure they were given an opportunity to shower and have breakfast before being discharged.

#### Light Exposure

The four white light conditions (Table 1) included a dim light (Dim), a light similar in intensity to the artificial light at home (Blue-Intermediate), a blue-enhanced light with intensity similar to artificial light at home (Blue-Enhanced) and a blue enhanced light brighter than the light at home (Bright Blue-Enhanced). The lights were administered in a counterbalanced, pseudo-randomized manner (one of two sequences) using purpose-designed boxes (Philips Lighting; Figure 1) which allowed us to switch between the light conditions. 

**Table 1:** Characteristics of the White Light Mixtures.

**Light Condition**	**Lamp Type (Color Temperature)**	**Illuminance (Lux)**	**Melanopsin Weighted Photon Density (photons/m^2^/s)**	**L-Cone Weighted Photon Density (photons/m^2^/s)**	**M-Cone Weighted Photon Density (photons/m^2^/s)**	**S-Cone Weighted Photon Density (photons/m^2^/s)**	**Photon Density (photons/m^2^/s)**
Dim	2700k (2592K)	19	2.09 x 10^16^	9.27 x 10^16^	5.92 x 10^15^	8.85 x 10^15^	1.60 x 10^17^
Blue-Intermediate	2700k (2529K)	200	1.87 x 10^17^	9.78 x 10^17^	6.21 x 10^17^	5.62 x 10^16^	1.55 x 10^18^
Blue-Enhanced	17000K (7717K)	195	6.23 x 10^17^	8.45 x 10^17^	7.19 x 10^17^	4.25 x 10^17^	1.70 x 10^18^
Bright Blue-Enhanced	17000K (7280K)	750	2.32 x 10^18^	3.27 x 10^18^	2.78 x 10^18^	1.54 x 10^18^	6.45 x 10^18^

The White light mixtures were designed with intensity and spectral composition as detailed in the table. Illuminance values in Columns 3 represent the design specifications. Columns 4, 5, 6, and 7 represent the light mixtures quantified according to the peak spectral sensitivities based on CVRL (http://www.cvrl.org) of the melanopsin (480 nm), L-cone (570 nm), M-cone (545 nm) and S-cone (445 nm) systems, respectively.

 At manufacture, light spectra and vertical illuminance at eye level were determined with a spectrometer (Spectrascan PR-704 with Spectrawin 2 software; Photo Research Inc.) and a handheld photometer (LMT Pocket Lux 2 meter) pointed at the rear inside wall of the box. Photon densities for each light condition were calculated by multiplying the Spectrascan measured photon densities/lux with the corresponding LMT lux readings (Table 1). On-site at the laboratory, irradiances (μW/cm^2^) for each light condition were determined with spectroradiometry recordings (HR2000, Ocean Optics, FL, USA) made in the wavelength range 344 - 782 nm with a sampling resolution of 0.23 nm and preceded by a dark calibration. 

### Activity and Light Exposure

 Actigraphy recording (1 minute resolution) in the field and laboratory were done with actiwatches (Actiwatch Spectrum, Philips Respironics) that measured wrist activity frequency, levels of broad spectrum white light, red (600-700nm), green (500-600 nm) and blue (400-500 nm) wavelengths. An accelerometer in the actiwatch measures the activity count while three independent color sensitive photodiodes measure illuminance (integrated input of the three sensors) and irradiance levels. Average light exposures were computed separately for the field and laboratory segments.

### Alertness

 Subjective sleepiness was assessed with a pencil and paper version of the KSS [27]. The KSS was administered before each cognitive testing session (KSS1) and at half-hourly intervals (KSS2) starting at the end of the first cognitive assessment (Figure 1, lower left rectangle). The two assessments were intended to track sleepiness (KSS1) and measure any change in sleepiness related to fatigue arising from intense mental effort, such as, after a series of cognitive tasks (KSS2). 

### Cognitive Performance

 Only pencil and paper tasks or computerized auditory tasks were used to avoid the confounding effect of light from the computer monitor. 

#### Sustained Attention

Simple response time tasks like the PVT are useful for examining circadian and homeostatic effects in sustained attention [23,28,36,37]. We used a 10-minute auditory variant of the task (referred to as PVT here). Participants responded with a mouse click as soon as they detected an auditory ‘beep’, which appeared after a random time interval between 2000 and 10,000 msecs. The primary measure was response time (RT) [37]. 

#### Cognitive Throughput

Numerical addition of digit pairs is commonly used to examine circadian and homeostatic effects in cognitive throughput [4,13,29]. We used a pencil and paper version in which the stimuli consisted of two-digit number pairs generated with a pseudo-random number generator. Participants had to mentally add as many such pairs as possible in a 2 minute period. No stimulus pair was repeated during or across laboratory segments for a given participant. The primary measures were the number of correct responses (accuracy) and attempted responses (speed). 

#### Working Memory

Verbal n-back tasks are popularly used for examining circadian and homeostatic effects in working memory [15,38]. In these tasks, the stimuli (letters) are presented in a rapid serial manner with the participant having to indicate whether or not the current stimulus matches a previous one. We used a 2-minute auditory variant of the verbal 1-back and 3-back tasks (inter-stimulus interval: 1000 milliseconds (ms); stimulus delay: 2000 ms). Each n-back sequence consisted of letters randomly chosen from a set of 9 consonants, and consisted of an equal number of “match” (‘yes’ response, 16 trials) and “non-match” (‘no’ response, 16 trials) trials. The dependant measures included RT, accuracy (number of correct responses) and aprime (index of accuracy based on Hits and false Alarms [38]). We used the standard practice of analyzing the ‘match’ and non-match’ trials, both, combined and separately, since the two types of trials are thought to involve different underlying mechanisms [15,39]. 

### Polysomnography (PSG)

Data were recorded with the Siesta 802 digital PSG system (Compumedics Limited, Victoria, Australia). An electroencephalography (EEG) montage based on the 10-20 system, consisting of F3-A2, F4-A1, C3-A2, C4-A1, O1-A2 and O2-A1 was used. Submental electromyogram (EMG), electro-cardiogram (ECG), Respiratory Effort (acclimation night only) and electro-occulogram (EOG) were also recorded. The EOG electrodes were placed at the outer canthi of each eye with the left and right electrodes referenced to the contra lateral mastoids A_1_ (left) and A_2_ (right). 

All signals were digitized on-line (16 bit AD converter; storage sampling rate at 256 Hz for EEG, EMG, EOG, ECG and 64 Hz for the Respiratory Effort). All recordings were visually scored on a 30-s epoch basis according to the AASM scoring criteria [40]. Sleep onset latency (SOL) and Latency to persistent sleep (LPS) were defined as the time (minutes) from lights off to the first epoch of NREM or REM sleep and the first consecutive 20 epochs of NREM or REM sleep, respectively. Latency to slow-wave sleep (SWSLAT) and REM sleep latency (REM LAT) were defined as the time (minutes) from SOL to the first epoch of stage N3 and REM sleep, respectively. The sleep stage at awakening was categorized as the most frequently occurring stage five minutes before lights-on [13]. 

### Statistical Analyses

All the data were analyzed using the statistical package SAS 9.1 (SAS Institute, Cary, North Carolina). 

 Our analyses focused on two aspects of the data: 1) the decrement in alertness and performance immediately upon awakening relative to baseline (before sleep), and 2) the change in alertness and performance during the four hours post-awakening. To assess the severity of sleep inertia we used two measures of effect size, Cohen’s d and Cohen’s f^2^. The former, derived from the means and standard deviations of the student’s t statistic, was used to assess the acute effect of sleep inertia [41]. It was based on the comparison of baseline performance (before sleep) with the first assessment after awakening in the morning. The latter, derived from the numerator and denominator degree of freedom of the F statistic from the mixed model ANOVA, was used to assess the dissipation in sleep inertia [42]. It was based on all the data from the morning testing sessions. 

 To characterize the effect of sleep inertia we focused on the data from the Dim light condition [4]. We used a general linear mixed-model ANOVA with an autoregressive [ar (1)] covariance structure, with ‘time relative to the start of the light exposure’ (time) as a repeated factor and as a fixed effect. To characterize the effect of the different white light conditions on sleep inertia, we used a similar model, but with both light condition and time as repeated factors and as fixed effects. For both analyses, ‘Subject’ was a random factor and the ‘randomization sequence of the light condition’ an additional factor to rule out order effects. Lastly, the baseline data (data from the evening session before the night sleep) was a covariate in the model. Normality of the residuals was checked for all the data sets and they did not deviate significantly from a normal distribution. Any data loss resulting in reduced sample sizes are stated in their corresponding sections in Results. 

## Results

 Our objective was to compare morning sleep inertia in alertness, attention, working memory and cognitive throughput and to determine whether sleep inertia exhibits an intensity- and spectrum-dependent sensitivity to light. The data presented in the figures and tables represent the actual means.

 First, our analysis of several EEG assessed sleep parameters (Table 2) allowed us to determine how well participants slept and whether sleep differed between the laboratory nights. A high sleep efficiency of above 89% during all sleep episodes and a lack of a significant difference (p >0.05) in sleep efficiency, SOL, SWSLAT, REM LAT between laboratory visits indicated that our participants slept the same and equally well on all four nights. We also analysed the most frequently occurring sleep stage during the 5 minutes before lights-on, as this has been shown to modulate the effect of sleep inertia [13]. Of the 44 awakenings, twice (5%) an individual was already awake at lights-on, 27 awakenings (61%) occurred from non-REM sleep and 15 (34%) from REM sleep (Table 3). 

**Table 2:** Sleep Parameters.

**Sleep Parameters**	**Dim (D) n=11**	**Blue-Intermediate (BI) n=11**	**Blue-Enhanced (BE) n=11**	**Bright Blue Enhanced (BBE) n=11**
Latency to Persistent Sleep (LPS; minutes)	14.00 + 2.16	17.82 + 8.17	12.59 + 4.22	23.40 + 10.40
Sleep Onset Latency (SOL; minutes)	11.09 + 2.05	12.50 + 6.70	11.55 + 4.24	21.20 + 10.44
Latency to Slow-Wave Sleep (SWSLAT; minutes)	13.41 + 1.02	15.68 + 4.01	13.00 + 0.95	13.25 + 1.39
REM Latency (REMLAT; minutes)	87.36 + 8.19	81.64 + 12.81	72.82 + 7.21	85.65 + 7.13
Total Sleep Time (TST; minutes)	356.55 + 10.57	364.73 + 8.35	364.86 + 8.91	350.60 + 11.83
Wake After Sleep Onset (WASO; minutes)	22.36 + 9.49	12.77 + 4.32	13.59 + 4.87	18.20 + 6.85
Wakefulness	22.36 + 9.49	11.55 + 4.16	13.59 + 4.87	17.15 + 6.89
Stage 1 (minutes)	31.91 + 2.78	34.00 + 2.47	34.36 + 3.16	31.20 + 4.85
Stage 2 (minutes)	161.91 + 9.37	171.95 + 6.55	169.55 + 8.21	167.10 + 8.45
Stage 3 (minutes)	94.18 + 8.18	86.00 + 5.83	89.91 + 7.08	88.25 + 4.83
Non-Rapid Eye Movement (NREM; minutes)	288.00 + 9.90	291.95 + 7.66	293.82 + 8.45	286.55 + 9.83
Rapid Eye Movement (REM; minutes)	68.55 + 7.04	72.77 + 5.15	71.05 + 5.69	64.05 + 6.00
Sleep Efficiency (SEFF %)	91.43 + 2.71	93.52 + 2.14	93.55 + 2.29	89.91 + 3.03

The data are actual means ± SEM (minutes) from the data. There were no significant differences in any of these sleep parameters between the laboratory sleep episodes.

**Table 3:** Percentage of Awakenings from a Specific Sleep Stage during the 5 minutes before Lights On.

**Stage at Wake**	**Dim (D) n=11**	**Blue-Intermediate (BI) n=11**	**Blue-Enhanced (BE) n=11**	**Bright Blue Enhanced (BBE**)** n=11**
Wakefulness	0%	10%	0%	10%
Non-Rapid Eye Movement (NREM; minutes)	55%	63%	55%	72%
Rapid Eye Movement (REM; minutes)	45%	27%	45%	18%

**Figure 2:**
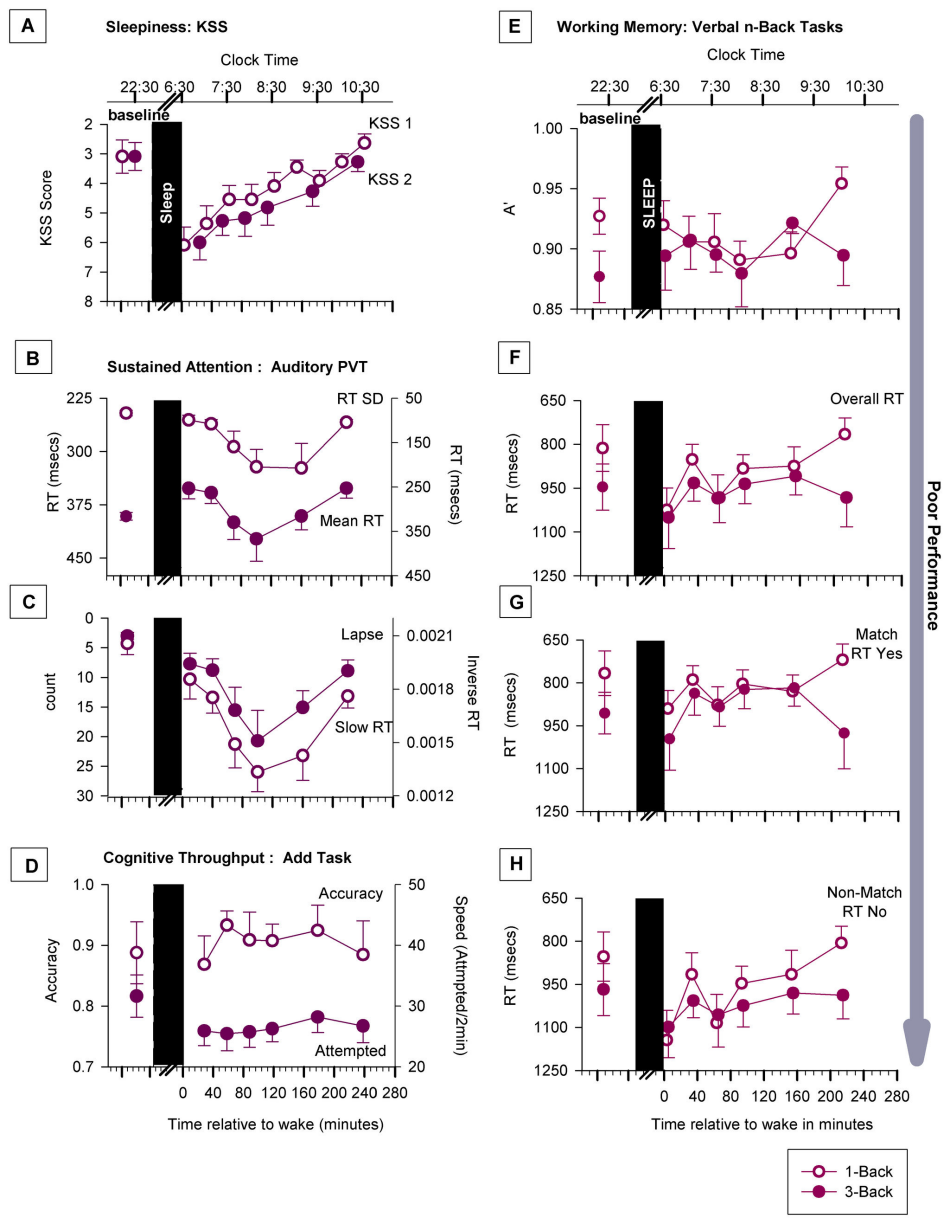
Sleep inertia in subjective alertness and cognition. Values shown here are the actual means +/- 1 SEM from the data. Baseline performance is shown to the left of the sleep episode while sleep inertia performance is shown to the right of it. (A) The effect of sleep inertia on subjective alertness as measured by the Karolinska Sleepiness Scale (KSS). The open circles depict alertness levels before a cognitive assessment (KSS1), while the closed circles depict half-hourly levels starting with the end of the first sleep inertia cognitive assessment (KSS2). (B) Mean response time (RT) and the variability of the mean RT on the PVT. (C) Lapses (number of RT’s > 500 msecs) and the inverse mean of the slowest 10% of RT’s on the PVT. (D) Cognitive throughput as measured with accuracy (percentage correct responses during a 2 minute period) and speed (number of responses during a two minute period) in the add task. Panels E, F, G and H show the effect of sleep inertia on working memory as measured by the 1-back and 3-back tasks. (E) Aprime on the 1-back and 3-back tasks. (F) Average RT on the match and non-match combined in the 1-back and 3-back tasks. (G) RT on the match trials in the two tasks. (H) RT on the non-match trials in the two tasks.

 To examine sleep inertia in alertness, we analysed the KSS1 and KSS2 scores (Figure 2A; n = 11). A paired t-test between baseline and the first post-sleep assessment revealed a significant difference (p<0.01) in both KSS scores such that sleepiness immediately after awakening were higher than at baseline. Thereafter, it declined over a two-hour period as indicated by a significant effect of time (KSS1: p<0.0001; KSS2: p=0.02) in the ANOVA, and, a significant difference (p<0.05) in the paired comparisons between the first post-sleep score and the remaining scores (Figure 2A). There was no effect of sleep stage at awakening in the scores.

 To examine sleep inertia in attention, we analysed RT, the standard deviation of the RT, the inverse of the slowest 10% RT and the number of lapses (Figure 2B and C) from the auditory PVT task (n = 11). A paired t-test between baseline and the first post-sleep assessment revealed a significant difference in speed (mean RT: p<0.01; slow RT: p=0.05) and lapses (p=0.01), with two and half times as many lapses at awakening when compared to baseline (3 + 0.59 to 7.7 + 1.79). The effect of sleep inertia dissipated over the course of four hours as indicated by a significant effect of time (mean RT: p=0.01; standard deviation of RT: p = 0.053; slow RT: p< 0.01; lapses: p < 0.01) in the ANOVA. However, this dissipation exhibited a ‘U’ shaped pattern, similar to other reports in the literature [11,17], in that performance first worsened before it recovered (Figure 2B and 2C). Note the more than fivefold increase in lapses (20.27 + 5.18; Figure 2C) for up to 2.5 hours after awakening. There was no effect of sleep stage at awakening this task.

 To examine sleep inertia in cognitive throughput, we analysed number of attempted responses (speed) and percentage of correct responses (accuracy) in the add task (Figure 2D; n = 11). A paired t-test between baseline and the first post-sleep assessments yielded a significant difference in speed (p<0.01; 30.6 + 3.5 vs. 25.9 + 2.4) but not in accuracy (p=0.78). The ANOVA yielded a significant effect of time on speed and accuracy (p=0.052), such that performance recovered to baseline levels by the end of four hours (Figure 2 D). The effect of sleep inertia in this task, however, was very modest. There was no effect of sleep stage at awakening this task.

 To examine sleep inertia in working memory we analysed aprime (the accuracy index) and RT (combined and separately for ‘match’ and non-match’ trials) in the 1-back and 3-back tasks (Figure 2, E-H; n = 11). Overall, speed more than accuracy was affected by sleep inertia in both tasks. A paired t-test between baseline and first post-sleep ‘aprime’ (Figure 2E) indicated no significant difference either the 1-back (0.92 + 02 vs. 00.92 + 02; p = 0.8) or 3-back (0.88 + 02 vs. 00.89 + 03 p = 0.6) task. The ANOVA results were similar in that there was no effect of time on aprime in either task.

 In contrast, the comparison of the baseline and first post-sleep RT (for the combined ‘match and ‘non match’ trials; Figure 2F), revealed a significant increase from baseline in the 1-back task (211 msec; p = 0.02), but, not in the 3-back task (104 msec; p = 0.09). When the ‘match’ and ‘non-match’ trials (Figure 2G and 2H, respectively) were analyzed separately, there was a significant increase first post-sleep RT from baseline only for the ‘non-match’ trials (1-back: 290 msecs, p=0.01; 3-back: 130 msecs, p=0.03). The ANOVA results were similar. The analyses of the combined ‘match’ and ‘non-match’ RT revealed significant effect of time in the 1-back task (p<0.001), but not in the 3-back task (p=0.5). While responses speeded up progressively over the four hours in the 1-back task, they remained unchanged in the 3-back task (Figure 2F). A separate analysis of the ‘match’ and ‘non-match’ showed a significant effect of time only on the ‘non-match’ trials in the 1-back task (p<0.001); there was a 338 msec decrease in RT by the end the four hour sleep inertia testing period. A third ANOVA, in which n-back level (that is including data from both tasks) was a fixed effects factor, indicated no significant time by n-back level interaction (p>0.05). There was no effect of sleep stage at awakening in performance in either task.

**Figure 3:**
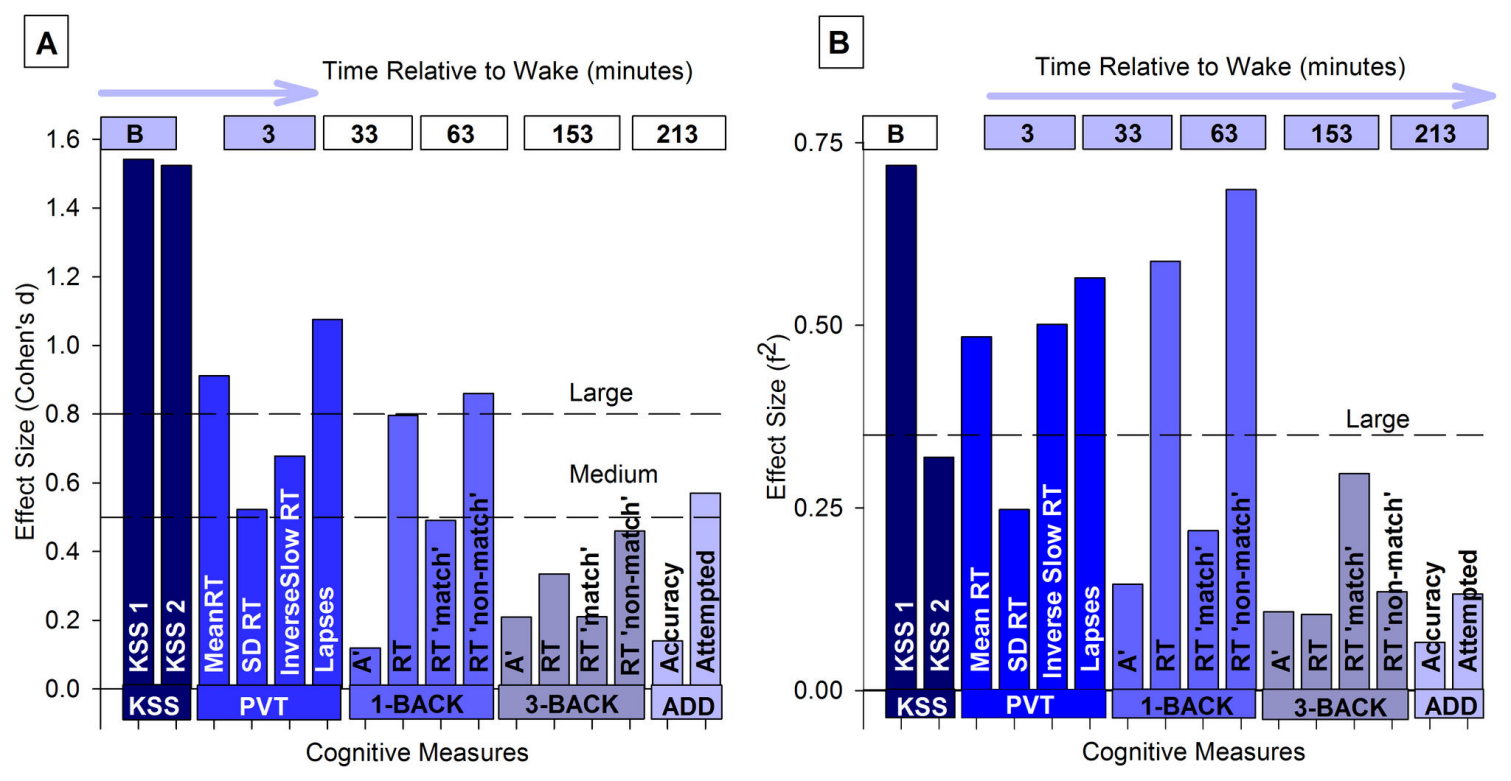
Effect Sizes. (A) A comparison of the acute effect of sleep inertia across the cognitive processes using Cohen’s d, computed with the mean and standard deviations on a student’s t-test between baseline (2 hours before bed-time) and initial sleep inertia assessments. The magnitude effect of sleep inertia appeared stronger alertness and in tasks primarily driven by lower order processes such as attention. (B) A comparison of the dissipation of sleep inertia across cognitive processes using implied f^2^ computed with the numerator and denominator degrees of freedom of the F statistic from the mixed-model ANOVA. The effect of sleep inertia appeared stronger in alertness and performance in tasks primarily mediated by attention.

 To examine the differential sensitivity of cognitive processes to sleep inertia we computed Cohen’s d (Figure 3A) and f^2^ (Figure 3B). First, the Cohen’s d was largest in the KSS scores compared to measures from the other tasks. Second, Cohen’s D was smaller in measures from the addition task and 3-back tasks in comparison to auditory RT task and the 1-back task. Third, regardless of task, the effect of sleep inertia was stronger in speed than in accuracy. The effect size based on Cohen’s f^2^ was again largest for the KSS scores, with performance in the addition and 3-back tasks showing smaller effect sizes in comparison to performance in the auditory PVT task and 1-back tasks. Again, the Cohen’s f^2^ was larger in speed than in accuracy. Thus, performance in simpler tasks appeared to be more affected by sleep inertia than performance in more demanding tasks.

**Figure 4:**
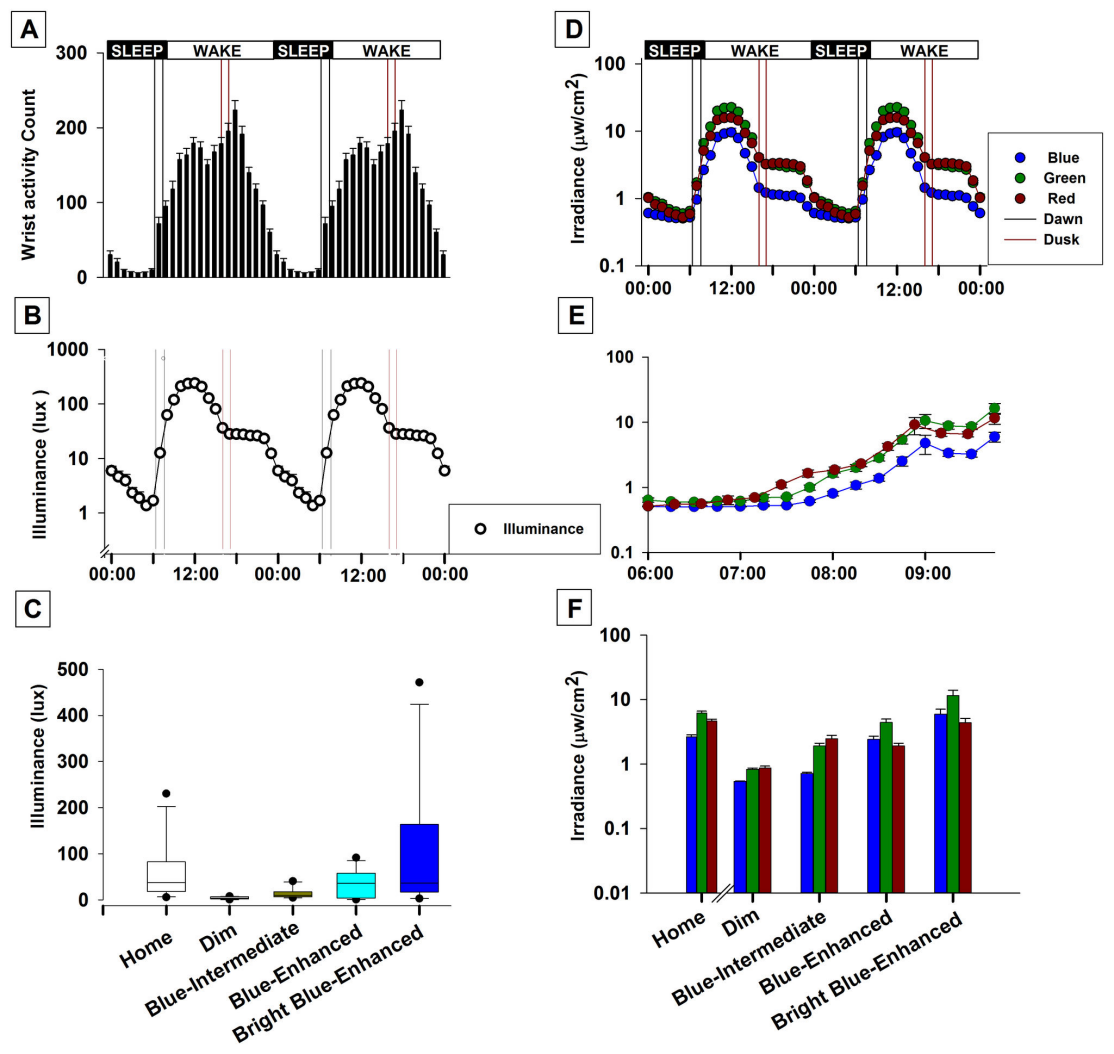
Comparison of the illuminance and spectral profiles of the experimental white-light mixtures and the white-light at home. (A) Double plot of the average 24-h profile of activity (upper plot) and Illuminance (lower plot) in the home environment of the participants The range of dawn and dusk during the study period ([October- December inclusive] 51°14'07.44"N latitude) are shown in the lower plot as black and red vertical reference lines, respectively. The black and red vertical reference lines represent the range of dawn and dusk respectively. Each data point in the two plots is an hourly average of 1 minute sampling, first computed for each participant and then averaged across the participants. (B) Double plot of the average 24-h profile of illuminance in the home environment of the participants. The black and red vertical reference lines represent the range of dawn and dusk respectively (C) Average morning illuminance while living at home and during the light exposure laboratory sessions. The Box-Plots show the 5^th^, 25^th^, median, 75^th^ and 90^th^ percentiles. (D) Double plot of the average 24-h profile of irradiance in the home environment of the participants. The black and red vertical reference lines represent the range of dawn and dusk respectively. (E) The average irradiance in the home environment of the participants just before wake time and during the morning hours corresponding to the laboratory light exposure session. (F) Average red, green and blue irradiance during the evening hours while living at home and during the laboratory light exposure sessions.

 The actigraphy measures were used to examine natural rest-activity and light-exposure patterns, as well as compare our experimental white light conditions with morning light at home (Figure 4; n =11). The data indicated that participants were active during the day and slept at night (Figure 4A). This corresponded with the 24-hour light exposure pattern which showed that illuminance was highest during the daytime, dropping sharply around dusk and stabilizing thereafter until the sleep onset (Figure 4B). Illuminance levels stayed low during sleep followed by a shallow rise around dawn which steepened sharply after wake time. These changes in illuminance were accompanied by corresponding changes in the blue, green and red wavelength light (Figure 4D and E). There was a sharp rise red, green and blue wavelength light after wake time, although the blue levels remained lower relative to the red and green levels, probably reflecting exposure to artificial light. 

 The four laboratory light conditions are summarized in Figure 4 (C and F). Mean illuminance levels (mean ± SEM: 58 ± 19.99 lux) at home in the morning were well above the Dim condition, although rather variable (range: 230 - 5.4 lux). The mean irradiance level in the blue light measured at home (2.64 ± 0.2 µw/cm^2^) was well above the blue irradiance of the Blue-Intermediate light (0.72 ± 0.3 µw/cm^2^) and well below the Blue-Enhanced light (5.92 ± 1.17µw/cm^2^). We note that there were significant differences in body position (and variations in this position) between the two environments. While participants had to sit relatively still with their wrists placed on the table during the light exposure, they would not have done so home. We also report specifications for the experimental light conditions derived from the photopic sensitivity and melanopsin based sensitivity curves (Table 1). 

**Figure 5:**
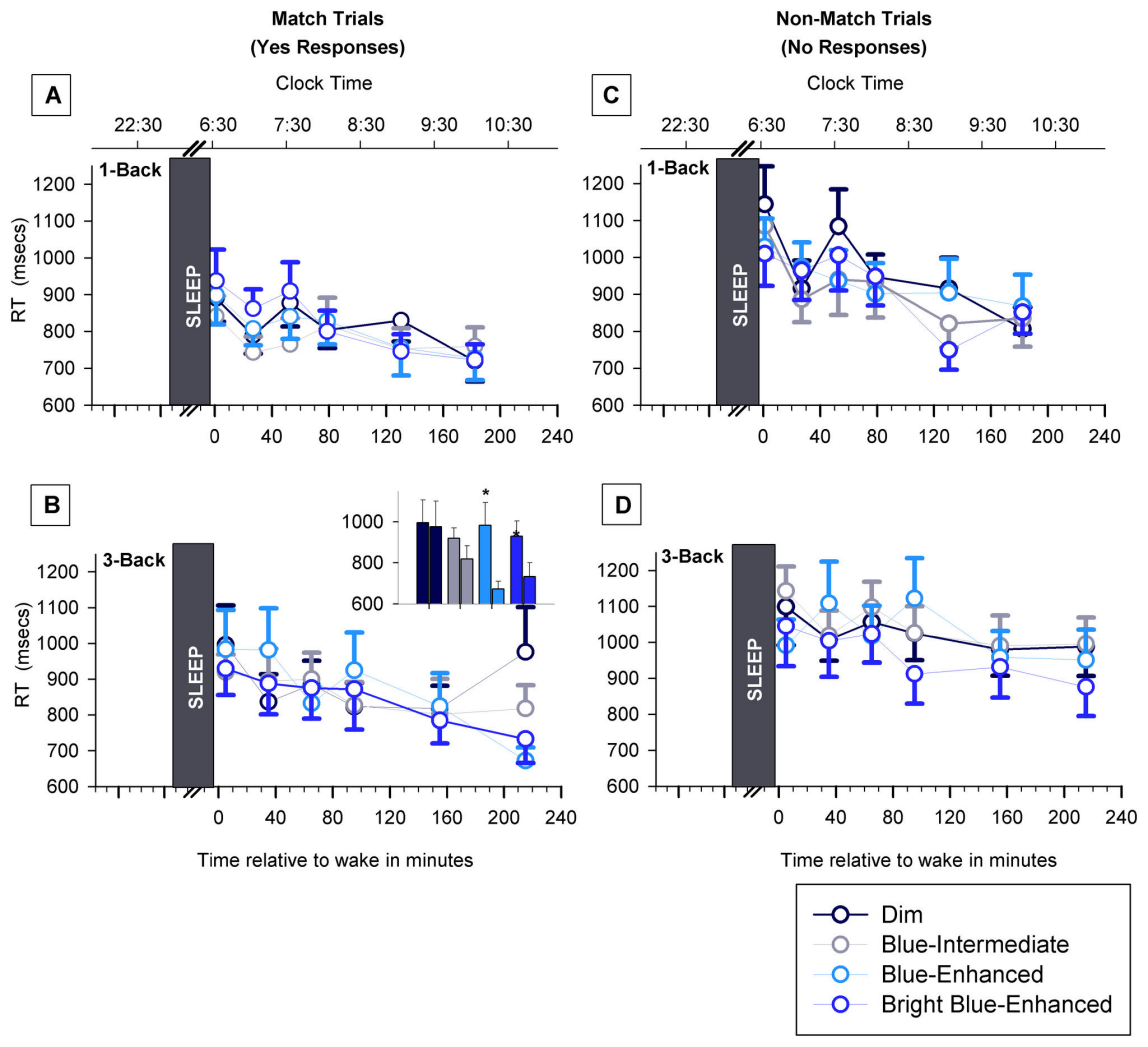
The effect of white light conditions on sleep inertia in working memory. Values shown here are the actual means +/- 1 SEM from the data. (A) RT on the match trials in the 1-back task. (B) RT on the match trials in the 3-back task. (C) RT on the non-match trials in the 1-back task. (D) RT on the non-match trials in the 3-back task. There was a significant light x time interaction only on the match trials in the 3-back task, as shown in the inset in panel B. The inset figure shows the RT’s from the first and last sleep inertia assessments; a significant difference was found in the Blue-Enhanced and Bright Blue-Enhanced conditions in the 3-back task.

 These different light conditions were used to determine whether sleep inertia exhibits an intensity- and spectrum-dependent sensitivity to light such that it would be significantly reduced in blue-enhanced light. Our analyses indicated that a differential effect of our white light conditions on sleep inertia was minimal (Figure 5B). With the exception of RT in the 3-back task, there were no significant effects of light condition on KSS or any other performance measure. An analysis of combined ‘match’ and ‘non-match’ RT showed no effect of light condition (p=0.25) or a time x light condition (p=0.14) interaction in the 3-back task. But, the separate analysis of the ‘match’ and ‘non-match’ trials, revealed a significant effect of time x light condition interaction for the ‘match’ trials in this task (p< 0.01; Figure 5B). RT at the end of the four hours in the two Blue-Enhanced conditions was significantly faster than at waketime (Figure 5B, inset). Lastly, there was no significant interaction between sleep-stage (at awakening) and light condition. 

## Discussion

 The aim of current study was to examine the differential effect of sleep inertia on various cognitive processes and determine if blue-enhanced artificial light might be an effective countermeasure for it. First, the effect of morning sleep inertia was widespread and far from being short-lived, lasted well over two hours. Second, alertness was most strongly affected by sleep inertia followed by attention, working memory and cognitive throughput. Notably, the easier tasks were more affected than the more demanding 3-back task. Lastly, Blue-Enhanced light did not appear to be very effective counteracting sleep inertia; its positive impact was confined to the response speed on the 3-back task, a task with high executive loading. 

 Prior sleep modulates the effect of sleep inertia. Its severity appears to depend partly on the quality and duration of preceding sleep [9] [3,4], that is shorter the sleep, the worse the sleep inertia. While the study did not directly test this, the 6.5 hr laboratory sleep episode was intended to optimise the opportunity to measure the effect of sleep inertia on performance not only by reducing sleep duration, but also by minimising wakefulness before ‘lights on’, which compromises the assessment of sleep inertia. While the effect of sleep inertia was strong in alertness, it was rather modest on cognitive performance. Besides duration and quality, the stage of sleep at awakening also appears to modulate sleep inertia [10,12,15], such that it has been shown to be worse following awakening from non-REM sleep, although there are results to the contrary [4,13,43]. We did not find any effect of sleep-stage at awakening in our data. Our sleep inertia assessment was done following a nocturnal sleep episode while many of the studies reporting an influence of sleep stage at awakening involved a nap protocol. Our results are however consistent with studies that have examined sleep inertia after night time sleep [13,18]. Therefore, whether naps exacerbate the effect of sleep stage at awakening on sleep inertia needs to be further examined. 

 The fact that we struggle to function effectively after awakening suggests that alertness and cognitive functions take time to be fully restored. How quickly this recovery occurs appears to depend on a variety of factors related to outcome variables and experimental manipulations [4,8,10,17]. We found that alertness/sleepiness and performance in tasks primarily involving lower cognitive processes, such as attention, were more affected than performance in tasks involving higher cognitive processes such as working memory (Figure 3A). The transition from sleep to wake, conceptualized as a ‘switch-like’ mechanism involving several populations of sleep and wake-promoting neurons switching states offers a possible an explanation for this task difference in sleep inertia [44]. It is likely that not all populations of neurons switch instantly between sleep and wake states, rather the duration of this shift varies among them. Thus, the restoration of a cognitive process upon awakening may be related to how quickly the neurons, mediating the process, shift between sleep-wake states. Indeed, data from a recent study of regional Cerebral Blood Flow (rCBF) during awakening suggests that brain areas associated with consciousness or arousal recover immediately upon awakening, while those associated with higher cognitive processes do so more slowly [6].

 Our participants appeared to be fully cognizant of their impairment in alertness, as was evident in the effect of sleep inertia on KSS ratings. However, the accuracy of self-ratings following sleep inertia or sleep loss has been debated in the literature [45,46]. While our results are consistent with some reports in the literature [4,8,10,17], it is at odds with more recent studies showing that sleep inertia may impair self-ratings of alertness, performance and mood [43,47,48]. It must be noted that our sleep inertia assessment was done after a nocturnal sleep episode while in the three studies [43,47,48] it occurred after a nap. Moreover, the outcome measures in two of the studies constituted self-ratings of performance rather than sleepiness, although, recently, Groeger et al. [15] found an effect of sleep inertia on subjective ratings of performance. Clearly, even small methodological changes and circadian effects can yield substantive differences in sleep inertia in cognition. 

 Attention was one of the more strongly affected cognitive processes in our study. PVT performance in the morning showed that the slowest responses were significantly longer and lapses increased more than twofold when compared to baseline. Lapses or extremely long RT’s (>500 msecs) in tasks like the PVT affect our ability to detect critical stimuli quickly and effectively. They are considered the hallmark of attention impairment [28,49] and constitute a unique behavioral state in that brain activity pattern during a lapse is different from the pattern during a normal or fast response [49,50]. 

 While sleep inertia in attention was as expected, the time course of its dissipation was unusual. It first worsened for two hours before improving and even then did not recover to baseline levels (Figure 2 B and 2C). Two other studies that examined sleep inertia in PVT performance report a similar pattern [11,17]. The acute fatiguing effect of this task in combination with sleep inertia may have led to the non-linear pattern in recovery from the sleep inertia. Moreover, the monotonous nature of the task could result in boredom and/or a lack of motivation, both of which could have contributed to the unusual pattern in recovery [17]. The fact that sleep inertia is associated with increased lapses implies serious consequences in many work environments.

 Sleep inertia had a very modest effect on cognitive throughput, and speed rather than in accuracy of performance was affected [4,29]. The data in the literature are rather contradictory in this regard. While some studies report an effect on speed in the addition task [4,17], others report an effect on accuracy [9,13,16]. Overall, findings from these studies suggest that various factors such as circadian phase, duration of sleep and conditions prior to sleep e.g. sleep deprivation, and experimental manipulations may modulate the relative sensitivity of speed and accuracy of performance in numerical tasks. For instance, sleep inertia appears strongest immediately upon awakening. In most studies of sleep inertia, the addition task is the first and often the only one to be administered [4,9,13,16,17]. In contrast, we administered the addition task after the N-back tasks and the PVT, and this may have diminished the effect of sleep inertia on this task; given the sample size of 11 it was not possible to vary the order of the tasks between participants to test this hypothesis. 

 As with the addition task, the effect of sleep inertia on working memory was seen in speed and not accuracy of performance. Most notably, the sleep inertia was stronger in the 1-back task compared to the 3-back task (Figure 3). These results are counterintuitive and in contrast to Groeger et al. [15]. But key methodological differences between our study and theirs may explain the contrasting results. First, we used an auditory version of the task whereas Groeger et al. [15] used a visual version of the tasks. Secondly, our sleep inertia assessment occurred after a night of sleep, while theirs was done after a morning or an afternoon nap. Critically, they only found this effect following the afternoon nap, which may indicate a circadian modulation [13]. Lastly, it is worth noting that Lo et al. [38] found a stronger effect of acute total sleep loss as well as repeated partial sleep loss on easier N-back tasks in comparison to the more challenging task. Why sleep inertia and sleep loss affect the easier n-back task more strongly is unclear. 

 Although the effect of sleep inertia is well documented, few studies have investigated therapeutic strategies for it [8,18]. Here, we asked whether modifying the composition of artificial light by making it brighter or blue-enhanced might reduce morning sleep inertia. Because, during the winter, in the higher latitudes, people wake up in darkness and turn on artificial light a few minutes later, we chose to simulate this morning light experience at home [51]. Although similar in composition to the artificial light at home, the four experimental light conditions varied in intensity and/or spectral composition, specifically in blue wavelength (Figure 4). We expected that blue-enhanced light would reduce the impact of sleep inertia. 

Contrary to our expectation, the two blue-enhanced lights had no effect on alertness/sleepiness and most of the performance measures [8]. However, we did find an effect of blue-enhanced white light on the ‘non-match’ trials on the 3-back task. But, because this effect was small its clinical significance or practical relevance is unclear. Perhaps, if sleep inertia is distinct from the homeostatic process [3–5] then it may have a different spectral/intensity sensitivity to light. For instance, Werken et al. [8] and Giminez et al. [18] found a positive effect on subjective alertness, albeit with a ‘dawn simulation’ light and without sleep restriction to 6.5 hrs..

 In conclusion findings from this study have implications for many socially critical professions e.g. medicine, law-enforcement and transportation, where personnel are routinely woken up from their night-time sleep, having to respond quickly and effectively to emergency situations. Clearly a phenomenon such as sleep inertia is of serious concern in these professions and it would be of great benefit to explore strategies to reduce its detrimental impact. Whether light exposure, a popular countermeasure for seasonal affective disorder, circadian misalignment and sleep loss during shift work, can be therapeutically used with sleep inertia needs to be more carefully examined. Although the light conditions in our study were not effective against sleep inertia, they may provide some useful information for future research in this regard. 

## References

[1] Jeanneret PR, Webb WB (1963) Strength of Grip On Arousal from Full Nights Sleep. Percept Mot Skills 17: 759-761. doi:10.2466/pms.1963.17.3.759. PubMed: 14085101. 14085101

[2] Tassi P, Muzet A (2000) Sleep inertia. Sleep Med Rev 4: 341-353. doi:10.1053/smrv.2000.0098. PubMed: 12531174. 12531174

[3] Achermann P, Werth E, Dijk DJ, Borbely AA (1995) Time course of sleep inertia after nighttime and daytime sleep episodes. Arch Ital Biol 134: 109-119. PubMed: 8919196. 8919196

[4] Jewett ME, Wyatt JK, Ritz-De CA, Khalsa SB, Dijk DJ, Czeisler CA (1999) Time course of sleep inertia dissipation in human performance and alertness. J Sleep Res 8: 1-8. doi:10.1046/j.1365-2869.1999.00001.x. PubMed: 10188130. 10188130

[5] Klerman EB, St Hilaire M (2007) On mathematical modeling of circadian rhythms, performance, and alertness. J Biol Rhythms 22: 91-102. doi:10.1177/0748730407299200. PubMed: 17440211. 17440211

[6] Balkin TJ, Braun AR, Wesensten NJ, Jeffries K, Varga M, Baldwin P, Belenky G, Herscovitch P (2002) The process of awakening: a PET study of regional brain activity patterns mediating the re-establishment of alertness and consciousness. Brain 125: 2308-2319. doi:10.1093/brain/awf228. PubMed: 12244087. 12244087

[7] Dinges DF, Orne MT, Whitehouse WG, Orne EC (1987) Temporal placement of a nap for alertness: contributions of circadian phase and prior wakefulness. Sleep 10: 313-329. PubMed: 3659730. 3659730

[8] van de Werken M, Giménez MC, de VB, Beersma DG, Van Someren EJ, Gordijn MC (2010) Effects of artificial dawn on sleep inertia, skin temperature, and the awakening cortisol response. J Sleep Res 19: 425-435. doi:10.1111/j.1365-2869.2010.00828.x. PubMed: 20408928. 20408928

[9] Balkin TJ, Badia P (1988) Relationship between sleep inertia and sleepiness: cumulative effects of four nights of sleep disruption/restriction on performance following abrupt nocturnal awakenings. Biol Psychol 27: 245-258. doi:10.1016/0301-0511(88)90034-8. PubMed: 3254730. 3254730

[10] Bruck D, Pisani DL (1999) The effects of sleep inertia on decision-making performance. J Sleep Res 8: 95-103. doi:10.1046/j.1365-2869.1999.00150.x. PubMed: 10389091. 10389091

[11] Ferrara M, De Gennaro L (2000) The sleep inertia phenomenon during the sleep-wake transition: theoretical and operational issues. Aviat Space Environ Med 71: 843-848. PubMed: 10954363. 10954363

[12] Silva EJ, Duffy JF (2008) Sleep inertia varies with circadian phase and sleep stage in older adults. Behav Neurosci 122: 928-935. doi:10.1037/0735-7044.122.4.928. PubMed: 18729646. 18729646 PMC7673910

[13] Scheer FA, Shea TJ, Hilton MF, Shea SA (2008) An endogenous circadian rhythm in sleep inertia results in greatest cognitive impairment upon awakening during the biological night. J Biol Rhythms 23: 353-361. doi:10.1177/0748730408318081. PubMed: 18663242. 18663242 PMC3130065

[14] Roenneberg T, Wirz-Justice A, Merrow M (2003) Life between clocks: daily temporal patterns of human chronotypes. J Biol Rhythms 18: 80-90. doi:10.1177/0748730402239679. PubMed: 12568247. 12568247

[15] Groeger JA, Lo JC, Burns CG, Dijk DJ (2011) Effects of sleep inertia after daytime naps vary with executive load and time of day. Behav Neurosci 125: 252-260. doi:10.1037/a0022692. PubMed: 21463024. 21463024

[16] Ferrara M, De Gennaro L, Bertini M (2000) Time-course of sleep inertia upon awakening from nighttime sleep with different sleep homeostasis conditions. Aviat Space Environ Med 71: 225-229. PubMed: 10716166. 10716166

[17] Hofer-Tinguely G, Achermann P, Landolt HP, Regel SJ, Retey JV et al. (2005) Sleep inertia: performance changes after sleep, rest and active waking. Brain Res. Cogn Brain Res 22: 323-331. doi:10.1016/j.cogbrainres.2004.09.013. 15722204

[18] Giménez MC, Hessels M, van de Werken M, de VB, Beersma DG, Gordijn MC (2010) Effects of artificial dawn on subjective ratings of sleep inertia and dim light melatonin onset. Chronobiol Int 27: 1219-1241. doi:10.3109/07420528.2010.496912. PubMed: 20653451. 20653451

[19] Vandewalle G, Balteau E, Phillips C, Degueldre C, Moreau V et al. (2006) Daytime light exposure dynamically enhances brain responses. Curr Biol 16: 1616-1621. doi:10.1016/j.cub.2006.06.031. PubMed: 16920622. 16920622

[20] Vandewalle G, Maquet P, Dijk DJ (2009) Light as a modulator of cognitive brain function. Trends Cogn Sci 13: 429-438. doi:10.1016/j.tics.2009.07.004. PubMed: 19748817. 19748817

[21] Chellappa SL, Steiner R, Blattner P, Oelhafen P, Götz T, Cajochen C (2011) Non-visual effects of light on melatonin, alertness and cognitive performance: can blue-enriched light keep us alert? PLOS ONE 6: e16429. doi:10.1371/journal.pone.0016429. PubMed: 21298068. 21298068 PMC3027693

[22] Chellappa SL, Gordijn MC, Cajochen C (2011) Can light make us bright? Effects of light on cognition and sleep. Prog Brain Res 190: 119-133. doi:10.1016/B978-0-444-53817-8.00007-4. PubMed: 21531248. 21531248

[23] Schmidt C, Collette F, Leclercq Y, Sterpenich V, Vandewalle G et al. (2009) Homeostatic sleep pressure and responses to sustained attention in the suprachiasmatic area. Science 324: 516-519 PubMed: 19390047. 10.1126/science.1167337 19390047

[24] Raymann RJ, Van Someren EJ (2007) Time-on-task impairment of psychomotor vigilance is affected by mild skin warming and changes with aging and insomnia. Sleep 30: 96-103. PubMed: 17310870. 17310870 10.1093/sleep/30.1.96

[25] Wagner U, Degirmenci M, Drosopoulos S, Perras B, Born J (2005) Effects of cortisol suppression on sleep-associated consolidation of neutral and emotional memory. Biol Psychiatry 58: 885-893. doi:10.1016/j.biopsych.2005.05.008. PubMed: 16005438. 16005438

[26] Thorn L, Hucklebridge F, Esgate A, Evans P, Clow A (2004) The effect of dawn simulation on the cortisol response to awakening in healthy participants. Psychoneuroendocrinology 29: 925-930. doi:10.1016/j.psyneuen.2003.08.005. PubMed: 15177708. 15177708

[27] Akerstedt T, Gillberg M (1990) Subjective and objective sleepiness in the active individual. Int J Neurosci 52: 29-37. doi:10.3109/00207459008994241. PubMed: 2265922. 2265922

[28] Dinges DF, Pack F, Williams K, Gillen KA, Powell JW et al. (1997) Cumulative sleepiness, mood disturbance, and psychomotor vigilance performance decrements during a week of sleep restricted to 4-5 hours per night. Sleep 20: 267-277. PubMed: 9231952. 9231952

[29] Jewett ME, Kronauer RE (1999) Interactive mathematical models of subjective alertness and cognitive throughput in humans. J Biol Rhythms 14: 588-597. doi:10.1177/074873099129000920. PubMed: 10643756. 10643756

[30] Buysse DJ, Reynolds CF III, Monk TH, Berman SR, Kupfer DJ (1989) The Pittsburgh Sleep Quality Index: a new instrument for psychiatric practice and research. Psychiatry Res 28: 193-213. doi:10.1016/0165-1781(89)90047-4. PubMed: 2748771. 2748771

[31] Archer SN, Robilliard DL, Skene DJ, Smits M, Williams A, Arendt J, von SM (2003) A length polymorphism in the circadian clock gene Per3 is linked to delayed sleep phase syndrome and extreme diurnal preference. Sleep 26: 413-415. PubMed: 12841365. 12841365 10.1093/sleep/26.4.413

[32] Lázár AS, Slak A, Lo JC, Santhi N, von Schantz M et al. (2012) Sleep, diurnal preference, health, and psychological well-being: a prospective single-allelic-variation study. Chronobiol Int 29: 131-146. doi:10.3109/07420528.2011.641193. PubMed: 22324552. 22324552

[33] Akerstedt T, Hume K, Minors D, Waterhouse J (1994) The subjective meaning of good sleep, an intraindividual approach using the Karolinska Sleep Diary. Percept Mot Skills 79: 287-296. doi:10.2466/pms.1994.79.1.287. PubMed: 7991323. 7991323

[34] Monk TH, Flaherty JF, Frank E, Hoskinson K, Kupfer DJ (1990) The Social Rhythm Metric. An instrument to quantify the daily rhythms of life. J Nerv Ment Dis 178: 120-126. doi:10.1097/00005053-199002000-00007. PubMed: 2299336. 2299336

[35] Blatter K, Cajochen C (2007) Circadian rhythms in cognitive performance: methodological constraints, protocols, theoretical underpinnings. Physiol Behav 90: 196-208. doi:10.1016/j.physbeh.2006.09.009. PubMed: 17055007. 17055007

[36] Wyatt JK, Ritz-De CA, Czeisler CA, Dijk DJ (1999) Circadian temperature and melatonin rhythms, sleep, and neurobehavioral function in humans living on a 20-h day. Am J Physiol 277: R1152-R1163. 10516257 10.1152/ajpregu.1999.277.4.r1152

[37] Jung CM, Ronda JM, Czeisler CA, Wright KP Jr. (2011) Comparison of sustained attention assessed by auditory and visual psychomotor vigilance tasks prior to and during sleep deprivation. J Sleep Res 20: 348-355. doi:10.1111/j.1365-2869.2010.00877.x. PubMed: 20819145. 20819145 PMC3603691

[38] Lo JC, Groeger JA, Santhi N, Arbon EL, Lazar AS et al. (2012) Effects of partial and acute total sleep deprivation on performance across cognitive domains, individuals and circadian phase. PLOS ONE 7: e45987. doi:10.1371/journal.pone.0045987. PubMed: 23029352. 23029352 PMC3454374

[39] Chen YN, Mitra S, Schlaghecken F (2008) Sub-processes of working memory in the N-back task: an investigation using ERPs. Clin Neurophysiol 119: 1546-1559. doi:10.1016/j.clinph.2008.03.003. PubMed: 18448388. 18448388

[40] Iber C, Ancoli-Israel S, Chesson A, Quan S (2007) The AASM manual for the scoring of sleep and associated events: rules, terminology and technical specifications. Westchester, Illinois: American Academy of Sleep Medicine.

[41] Cohen J (1988) Statistical Power Analysis for the Behavioral Sciences. Hillsdale, NJ: Lawerence Earlbaum Associates.

[42] Van Dongen HP, Maislin G, Kerkhof GA (2001) Repeated assessment of the endogenous 24-hour profile of blood pressure under constant routine. Chronobiol Int 18: 85-98. doi:10.1081/CBI-100001178. PubMed: 11247116. 11247116

[43] Signal TL, van den Berg MJ, Mulrine HM, Gander PH (2012) Duration of sleep inertia after napping during simulated night work and in extended operations. Chronobiol Int 29: 769-779. doi:10.3109/07420528.2012.686547. PubMed: 22734577. 22734577

[44] Saper CB, Fuller PM, Pedersen NP, Lu J, Scammell TE (2010) Sleep state switching. Neuron 68: 1023-1042. doi:10.1016/j.neuron.2010.11.032. PubMed: 21172606. 21172606 PMC3026325

[45] Kaida K, Takahashi M, Akerstedt T, Nakata A, Otsuka Y et al. (2006) Validation of the Karolinska sleepiness scale against performance and EEG variables. Clin Neurophysiol 117: 1574-1581. doi:10.1016/j.clinph.2006.03.011. PubMed: 16679057. 16679057

[46] O'Donnell D, Silva EJ, Münch M, Ronda JM, Wang W, Duffy JF (2009) Comparison of subjective and objective assessments of sleep in healthy older subjects without sleep complaints. J Sleep Res 18: 254-263. doi:10.1111/j.1365-2869.2008.00719.x. PubMed: 19645969. 19645969 PMC2975570

[47] Kubo T, Takahashi M, Takeyama H, Matsumoto S, Ebara T et al. (2010) How do the timing and length of a night-shift nap affect sleep inertia? Chronobiol Int 27: 1031-1044. doi:10.3109/07420528.2010.489502. PubMed: 20636214. 20636214

[48] Asaoka S, Masaki H, Ogawa K, Murphy TI, Fukuda K, Yamazaki K (2010) Performance monitoring during sleep inertia after a 1-h daytime nap. J Sleep Res 19: 436-443. doi:10.1111/j.1365-2869.2009.00811.x. PubMed: 20374446. 20374446

[49] Chee MW, Tan JC, Zheng H, Parimal S, Weissman DH et al. (2008) Lapsing during sleep deprivation is associated with distributed changes in brain activation. J Neurosci 28: 5519-5528. doi:10.1523/JNEUROSCI.0733-08.2008. PubMed: 18495886. 18495886 PMC6670628

[50] Weissman DH, Roberts KC, Visscher KM, Woldorff MG (2006) The neural bases of momentary lapses in attention. Nat Neurosci 9: 971-978. doi:10.1038/nn1727. PubMed: 16767087. 16767087

[51] Santhi N, Thorne HC, van der Veen DR, Johnsen S, Mills SL et al. (2012) The spectral composition of evening light and individual differences in the suppression of melatonin and delay of sleep in humans. J Pineal Res 53: 47-59. doi:10.1111/j.1600-079X.2011.00970.x. PubMed: 22017511. 22017511

